# Exploring the Triangle Between Oxidative Stress, Advanced Glycation End Products and Dental Caries in the Context of Diet and Lifestyle

**DOI:** 10.3390/nu18060923

**Published:** 2026-03-14

**Authors:** Sebastian Candrea, Alessio Danilo Inchingolo, Alexandrina Muntean, Ioana-Roxana Bordea, Anida-Maria Băbțan, Cosmina Ioana Bondor, Marian Tăulescu, Gabriela Roman, Georgeta Inceu, Adina Bianca Boșca, Francesco Inchingolo, Laura Ferrante, Angelo Michele Inchingolo, SALIVAGES Project Group, Gianna Dipalma, Friederike Manig, Michael Hellwig, Thomas Henle, Aranka Ilea

**Affiliations:** 1 Department of Oral Rehabilitation, Faculty of Dentistry, “Iuliu Hațieganu” University of Medicine and Pharmacy, 400012 Cluj-Napoca, Romania; sebastian.candrea@umfcluj.ro (S.C.); babtan.anida@umfcluj.ro (A.-M.B.); aranka.ilea@umfcluj.ro (A.I.); 2 Department of Paediatric Dentistry, “Iuliu Hatieganu” University of Medicine and Pharmacy, 400012 Cluj-Napoca, Romania; alexandrina.muntean@umfcluj.ro; 3 Department of Interdisciplinary Medicine, School of Medicine, University of Bari “Aldo Moro”, 70124 Bari, Italy; alessiodanilo.inchingolo@uniba.it (A.D.I.); francesco.inchingolo@uniba.it (F.I.); lauraferrante79@virgilio.it (L.F.); angelomichele.inchingolo@uniba.it (A.M.I.); gianna.dipalma@uniba.it (G.D.); 4 Department of Medical Informatics and Biostatistics, Faculty of Medicine, “Iuliu Hațieganu” University of Medicine and Pharmacy, 400337 Cluj-Napoca, Romania; cbondor@umfcluj.ro; 5 Department of Veterinary Pathology, Faculty of Veterinary Medicine, University of Agricultural Sciences and Veterinary Medicine of Cluj-Napoca, 400372 Cluj-Napoca, Romania; marian.taulescu@usamvcluj.ro; 6 Department of Diabetes, Nutrition and Metabolic Diseases, Faculty of Medicine, “Iuliu Hațieganu” University of Medicine and Pharmacy, 400337 Cluj-Napoca, Romania; groman@umfcluj.ro; 7 Department of Pathophysiology, Faculty of Medicine, “Iuliu Hațieganu” University of Medicine and Pharmacy, 400337 Cluj-Napoca, Romania; inceu.victoria@umfcluj.ro; 8 Department of Histology, Faculty of Medicine, “Iuliu Hațieganu” University of Medicine and Pharmacy, 400337 Cluj-Napoca, Romania; bianca.bosca@umfcluj.ro; 9 Department of Life Science, Health, and Health Professions, Link Campus University Italy Macerata, 00165 Rome, Italy; 10 Department of Biomedical, Surgical and Dental Sciences, Milan University, 20122 Milan, Italy; 11 Department of Chemistry and Food Chemistry, Technische Universität Dresden, 01307 Dresden, Germany; friederike.manig@tu-dresden.de (F.M.); michael.hellwig@tu-dresden.de (M.H.); thomas.henle@tu-dresden.de (T.H.)

**Keywords:** dental caries, oxidative stress, advanced glycation end products (AGEs), dietary habits, lifestyle

## Abstract

**Background/Aim:** Dental caries is a multifactorial disease influenced by dietary habits, lifestyle factors, and host biochemical processes. Oxidative stress and advanced glycation end products (AGEs) have been implicated in oral and systemic pathophysiology, but their combined association with caries experience remains unclear. This study aimed to evaluate the relationships between caries indices, diet, smoking, oxidative stress markers, and AGEs in adults. **Materials and Methods:** A cross-sectional study was conducted on adults enrolled in the SALIVAGES project (2018–2020). Dental status was assessed using the DMFT index. Dietary habits and smoking status were recorded using a validated questionnaire. Oxidative stress parameters (TAC, TOS, OSI, NO, MDA, total thiols) and AGEs (FruLys, MG-H1, CML, CEL, Pyr, Arg, Lys) were quantified in saliva and plasma. Associations were analyzed using correlation tests and multivariable regression models (α = 0.05). **Results:** The mean DMFT was 21.89 ± 7.13, with missing teeth predominating. Caries experience was significantly associated with oxidative stress, AGEs, diet, and lifestyle. Higher decay scores were associated with increased NO and total thiols and reduced antioxidant capacity. Several salivary AGE-related biomarkers (FruLys, MG-H1, CML, and CEL) were negatively associated with the decay index. Sugary beverages, refined carbohydrates, pastries, and donuts were strongly positively associated with the decay index, whereas wholemeal bread showed an inverse association with caries indices. Smoking was independently associated with higher decay and DMFT values, corresponding to an approximately three-unit higher DMFT score. **Conclusions:** Caries experience in adults is associated with dietary, lifestyle, and biochemical factors. Sugar intake and smoking showed the strongest associations with caries indices, while oxidative stress parameters and selected salivary AGE-related biomarkers showed weaker but significant inverse associations with decay. These findings support preventive strategies targeting diet quality, smoking cessation, and redox balance to reduce oral disease burden.

## 1. Introduction

Nutrition and oral health are fundamental determinants of overall health and healthy aging. According to the World Health Organization and the FDI World Dental Federation, oral health is a multidimensional construct encompassing physical, functional, and psychosocial domains, including the ability to speak, chew, swallow, and express emotions without pain or disease [1,2]. Dental caries remains the most prevalent oral disease worldwide, affecting all age groups [3,4]. Its development is multifactorial, resulting from complex interactions among biological, environmental, social and behavioral determinants [4,5,6,7]. Established risk factors include free-sugar intake, salivary characteristics, fluoride exposure, and the presence of cariogenic microorganisms, alongside broader socio-demographic and behavioral influences [8,9,10]. Despite global preventive strategies emphasizing sugar reduction, caries continues to represent a substantial public health burden, particularly in adults, where cumulative disease experience is often reflected by high Decay Missing Filled Teeth (DMFT) index values and tooth loss [11,12,13,14].

Beyond its classical microbial and dietary determinants, dental caries may also be associated with broader metabolic and biochemical processes. Saliva exerts a secondary yet significant influence on caries development, acting as the biological medium responsible for mineral exchange between calcified dental tissues and the oral environment. In addition to this role, saliva displays immunological and enzymatic functions and represents a valuable diagnostic fluid for monitoring both local and systemic conditions [15,16]. A variety of glycation- and oxidation-related biomarkers can be detected in saliva, offering valuable insight into biochemical processes that may accompany oral health and disease. Salivary antioxidant enzymes are an important component of the oral antioxidant defense system and may modulate bacterial activity involved in the pathogenesis of dental caries and periodontal disease [17,18]. Oxidative stress arises when the equilibrium between reactive oxygen species (ROS) and antioxidant defenses is disrupted. Excessive ROS activity damages nucleic acids, lipids, and proteins, ultimately promoting tissue degeneration and cell death. Once antioxidant capacity is exceeded, ROS may alter cellular metabolism, disturb oral biofilm stability, and interfere with hormonal regulation [19]. A systemic theory of caries development proposed by Tóthová et al. suggests that ROS-induced alterations in parotid hormone secretion could be associated with increased dentin susceptibility to acid challenge. Moreover, carious lesions may themselves influence salivary antioxidant activity. Reduced salivary melatonin levels have been observed in patients with high caries susceptibility; considering that melatonin is a potent antioxidant capable of neutralizing malondialdehyde (MDA), a marker of lipid peroxidation, its depletion may further compromise oral defense mechanisms [20,21]. Antioxidant activity is also influenced by multiple systemic and local factors, such as age, sex, overall health status, and the presence or absence of dental restorations [22,23].

Although numerous studies have explored the association between dental caries, antioxidant capacity, and oxidative stress markers, the evidence remains inconclusive. The conflicting results reported in the literature may reflect the complex involvement of antioxidant defense mechanisms in the onset and progression of oral diseases [24,25]. Therefore, the present study aims to assess the relationships between patients’ caries experience, bio-humoral oxidative stress markers and advanced glycation end products, and lifestyle and alimentary habits.

## 2. Materials and Methods

### 2.1. Study Design and Ethical Considerations

This cross-sectional observational study, conducted between 2018 and 2020 at the Department of Oral Rehabilitation, included patients enrolled in the SALIVAGES project and was designed and reported in accordance with the Strengthening the Reporting of Observational Studies in Epidemiology (STROBE) guidelines. The protocol was approved by the University Ethics Committee (approval no. 93/8 March 2017). All participants provided written informed consent in accordance with the World Medical Association Declaration of Helsinki (revised 2013, Fortaleza) [26].

### 2.2. Patient Enrollment Protocol

Eligible participants were adults (≥18 years) referred for oral examination, with or without associated treatment needs. Demographic, clinical, and anthropometric data were recorded, including age, sex and body mass index (BMI). Venous blood samples were collected from the antecubital vein in sterile osmotic tubes [23]. Exclusion criteria were pregnancy, acute systemic infections at the time of examination, current antibiotic or anti-inflammatory therapy, and inability or refusal to provide informed consent.

During the recruitment period, eligible patients referred for routine oral examination were invited to participate consecutively, and those who provided written informed consent were included in the study.

A comprehensive exo-oral and oral examination was performed. Saliva samples were collected using citric acid-soaked sterile cotton rolls (Salivette^®^ Sarstedt AG & Co., Nümbrecht, Germany), chewed for 2 min. Samples were centrifuged at 1450 rpm for 7 min and aliquoted into sterile cryotubes (100, 200, and 500 μL). Saliva, plasma, and urine were stored at −20 °C until analysis.

Dietary habits were assessed through a comprehensive, structured questionnaire that included detailed items on multiple dimensions of eating behavior. Specifically, the instrument captured information on meal frequency, snacking patterns between meals, tendencies toward late-night eating, preferences for various types of beverages, levels of added sugar consumption, as well as general dietary practices and routines. The questionnaire’s psychometric properties—namely, its validity and reliability—had been rigorously evaluated and confirmed in a prior study conducted by Roman et al. [27].

To reduce measurement bias, all clinical examinations were performed by calibrated examiners using standardized procedures, and laboratory analyses were conducted using validated and standardized protocols. Consecutive sampling was used to minimize selection bias.

As this was a cross-sectional study, no follow-up procedures were performed.

### 2.3. DMFT Index Assessment

Dental caries experience was evaluated using the DMFT (Decayed, Missing, and Filled Teeth) index, calculated independently by two calibrated dentists based on patient records. For edentulous patients, the cause of tooth loss was recorded. In partially edentulous patients, the etiology of missing teeth was determined clinically: Multiple carious lesions were attributed to extractions due to caries complications, while extensive periodontal lesions indicated periodontal disease as the cause. When both conditions coexisted, tooth loss was attributed to carious pathology.

### 2.4. Periodontal Evaluation

Periodontal status was assessed by clinical probing and radiographic examination, and disease was classified according to stage and grade, following the Papapanou et al. classification [28].

### 2.5. Nitric Oxide (NO) Determination

Nitrite (NO_2_^−^) and nitrate (NO_3_^−^) concentrations, reflecting nitric oxide synthesis (NOx), were quantified using the Griess reaction. Plasma samples were filtered through 10 kDa cut-off membranes (Sartorius AG, Göttingen, Germany) and deproteinized with methanol/diethyl ether (3:1, *v*/*v*). After the reduction of nitrate to nitrite with VCl_3_ (100 μL, 8 mg/mL), Griess reagents—sulfanilamide (2%, 50 μL) and *N*-(1-naphthyl) ethylenediamine dihydrochloride (0.1%, 50 μL)—were added. Following 30 min incubation at 37 °C, absorbance was measured at 540 nm. Serum NOx concentrations were calculated using a sodium nitrite calibration curve and expressed as μmol/L.

### 2.6. Total Oxidative Status (TOS)

Plasma TOS was determined using a colorimetric method based on the oxidation of ferrous to ferric ions by reactive oxygen species under acidic conditions. Ferric ions were detected via reaction with xylenol orange. Hydrogen peroxide was used as the calibrator, and results were expressed as μmol H_2_O_2_ equivalents/L [29].

### 2.7. Total Antioxidant Capacity (TAC)

Total antioxidant capacity (TAC) was determined using a colorimetric assay based on hydroxyl radical generation via the Fenton reaction. The oxidation of o-dianisidine by hydroxyl radicals produces a colored complex, the intensity of which is inversely proportional to the antioxidant capacity of the sample. Plasma antioxidants suppressed this reaction, preventing color development. The assay was standardized with Trolox, and results expressed as mmol Trolox equivalents/L [30].

The oxidative stress index (OSI) was calculated as the TOS/TAC ratio, expressed in arbitrary units [31].

### 2.8. Malondialdehyde (MDA) Quantification

MDA, a marker of lipid peroxidation, was determined using the thiobarbituric acid reactive substances (TBARSs) method. Plasma (150 μL) was incubated with trichloroacetic acid (10%, 125 μL), EDTA (5 mM, 125 μL), sodium dodecyl sulfate (8%, 125 μL), and butylated hydroxytoluene (0.5 μg/mL, 10 μL). After mixing, the samples were incubated for 10 min at room temperature, followed by the addition of thiobarbituric acid (0.6%, 500 μL) and heating at 95 °C for 30 min. After cooling and centrifugation (10,000× *g*, 10 min), the absorbance of the supernatant was measured at 532 nm. MDA levels were calculated using a standard curve prepared with 1,1,3,3-tetraethoxypropane and expressed as nmol/mL plasma [32].

### 2.9. Advanced Glycation End Products (AGEs)

Salivary advanced glycation end products (AGEs), quantified as free glycated amino acids and expressed in ng/mL—namely, fructosyl-lysine (FruLys), methylglyoxal-derived hydroimidazolone-1 (MG-H1), *N*-ε-carboxymethyllysine (CML), *N*-ε-carboxyethyllysine (CEL), and pyrraline (Pyr)—as well as the corresponding unmodified amino acids arginine (Arg) and lysine (Lys), expressed in µg/mL, were measured according to the protocol described by Manig et al. [33]. Briefly, 500 μL saliva was mixed with 10 μL internal standard and 490 μL acetonitrile/methanol (70:30, *v*/*v*). After 10 min at 4 °C, samples were centrifuged (10,000× *g*, 10 min), the supernatant was evaporated under nitrogen, and residues were reconstituted in 90 μL NFPA (20 mM). For plasma, a 25 μL sample, a 10 μL internal standard, and a 400 μL acetonitrile/methanol (70:30, *v*/*v*) were mixed for deproteinization, incubated overnight at −18 °C, centrifuged, dried, and reconstituted in 20 mM NFPA. All samples were analyzed by liquid chromatography–tandem mass spectrometry (LC-MS/MS).

### 2.10. Data Analysis

The primary outcomes were the DMFT index and its components (Decay and Missing indices). Exposure variables included dietary consumption frequencies, smoking status, oxidative stress markers, and AGE-related biomarkers. Age was considered a potential confounder in multivariable models.

The minimal required sample size was calculated a priori using G*Power software (version 3.1.9.7). Considering that the primary objectives of the study were to evaluate correlations between the DMFT index, Decayed, Missing, and Filled Teeth indices and other investigated parameters, a medium effect size (r = 0.30), an alpha error probability of 0.05, and a minimum statistical power of 0.80 were assumed. Based on these parameters, the minimum required sample size was 84 participants. Anticipating that approximately 20% of participants might present missing data, an adjusted target sample size of 101 participants was estimated. Since a total of 210 participants were included in the present study, the a priori sample size requirement was considered fulfilled.

All study data were analyzed using IBM SPSS Statistics 25 and illustrated using Microsoft Office Excel/Word 2024. Qualitative variables were written as counts or percentages.

Quantitative variables were written as means with standard deviations or medians with interquartile ranges. The normality of the quantitative variables was assessed using the Shapiro–Wilk Test. Quantitative variables with non-parametric distribution were tested between groups using the Mann–Whitney U Test/Kruskal–Wallis H Test. Correlations between quantitative variables with non-parametric distribution were measured using Spearman’s rho correlation coefficients.

Univariable and multivariable regression models were used to examine associations between the DMFT index and its components (D, M, F) and the investigated parameters. Models were tested for linearity, independence of observations, residual normality, homoscedasticity, multicollinearity, and overall significance. The strength and direction of associations were quantified using beta coefficients with 95% confidence intervals. Multivariable models were obtained using the step-wise forward approach or the standard enter approach, in which significant parameters identified in univariable analyses were introduced into the models. Age was included as an adjustment variable in additional multivariable models to account for its potential confounding effect on cumulative caries experience.

Analyses were performed using a complete-case approach; participants with missing data for variables required in a given analysis were excluded from that specific analysis. Missing data were primarily related to specific biological sample categories (e.g., salivary or plasma oxidative stress and AGE determinations), as not all participants provided all types of biological samples or were included in every laboratory analysis panel. Clinical and questionnaire-based variables were largely complete. Overall, missingness ranged between 12% and 23% for biochemical parameters, while non-biochemical variables showed minimal missing data. The number of valid observations (N) for each variable is explicitly reported in the corresponding tables. No data imputation procedures were applied, and complete-case analysis was considered appropriate for this cross-sectional design.

The threshold considered for the significance level for all tests was considered to be α = 0.05.

## 3. Results

A total of 210 patients were enrolled in the study. Because not all participants had complete clinical, dietary or biochemical datasets, the number of valid observations (N) varied across variables. Consequently, each statistical test was performed on the maximum number of cases available for the respective parameter. Importantly, the sample sizes retained in multivariable regression models (N ranging between 152 and 188 depending on the model) exceeded the minimum required sample size calculated a priori (N = 101), thereby preserving adequate statistical power despite variable-specific missingness.

Data from Table 1 summarize the descriptive characteristics of the analyzed patients. Results show that most of the patients were women (64.6%), the average age was 50.55 ± 15.31 years (median = 53), with a mean BMI of 29.64 ± 6.94 (median = 28.75), and most patients had a stage III (29.4%) or stage IV (21.8%) periodontitis. Most of the patients reported dental hygiene twice/day (51%). According to the medical history, most of the patients had cardiovascular (39.8%), endocrine (16.3%), hepatic comorbidities (14.7%) or diabetes (13%).

Data from Table 2 show the distribution of the dental parameters analyzed in the study. The mean decay index was 5.2 ± 5.95, with a median of 4, the mean missing index was 15.47 ± 8.72, with a median of 15 and the mean DMFT index was 21.89 ± 7.13, with a median of 22.

Data from Table 3 show the plasma and salivary oxidative stress parameters analyzed in the study.

Data from Table 4 show the plasma and salivary AGE parameters analyzed in the study.

Data from Table 5 show the distribution of the patients according to smoking status. Overall, 42.1% of the patients were active smokers.

Data from Table 6 show the distribution of the patients according to eating habits. The results show the following.

For snacks, most patients reported daily consumption (40.5%);

For most of the food types—carbonated drinks with sweeteners (49.2%), carbonated drinks with sugar (55.1%), wholemeal flour bread (47.7%), croissants, muffins or biscuits (42.1%), pancakes or waffles (45.1%), cream (70.6%), milk chocolate (44.9%), dark chocolate (55.6%), chocolate bars (59.7%), donuts (50.8%), pies or puddings (46.2%), jam and honey (38.1%), ice cream (34.5%) and orange juice (46.4%)—most of the patients reported never consuming these products or consuming them less than once per month;

For white bread and other white flour products, most of the patients reported consumption 2–3 times/day (33.2%);

For cakes, most of the patients reported consumption 1–3 times/month (32.5%) or less than one time/month (32%);

For apples or pears, most of the patients reported consumption of 2–4 times/week (22.3%).

For most of the eating habits except for snacks, a score for consumption frequency was established, based on the type of response: 0 points for never/less than one time per month, 1 point for 1–3 times/month, 2 points for 1 time/week, 3 points for 2–4 times/week, 4 points for 5–6 times/week, 5 points for 1 time/day, 6 points for 2–3 times/day, 7 points for 4–5 times/day and 8 points for ≥6 times/day.

Data from Table 7 present Spearman correlations of the DMFT index, decay index and missing index with age. Results show that all correlations were significant (*p* < 0.05), and age was positively correlated with the DMFT index (*p* < 0.001, R = 0.284) and the missing index (*p* < 0.001, R = 0.649) and inversely correlated with the decay index (*p* < 0.001, R = −0.487).

**Figure 1:**
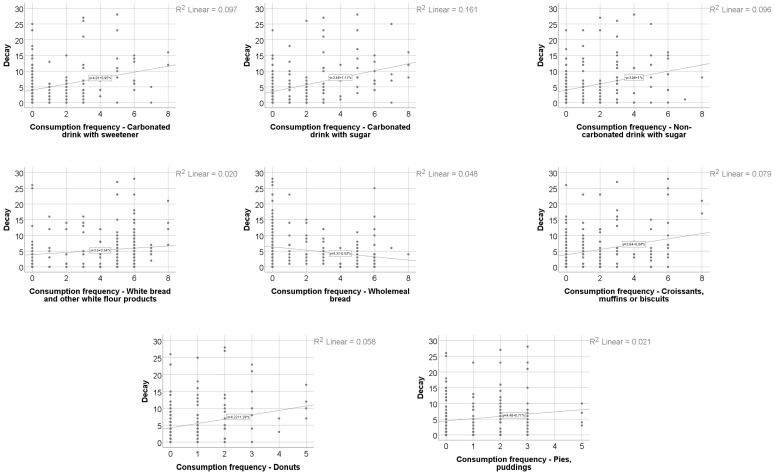
The correlation between caries index and dietary habits.

**Figure 2:**
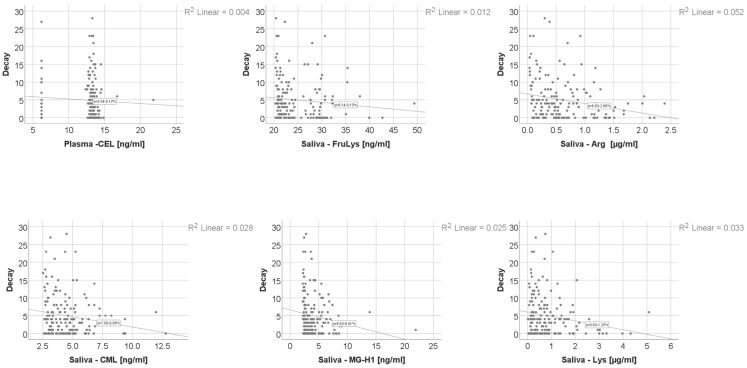
The correlation between caries index and AGEs.

**Figure 3:**
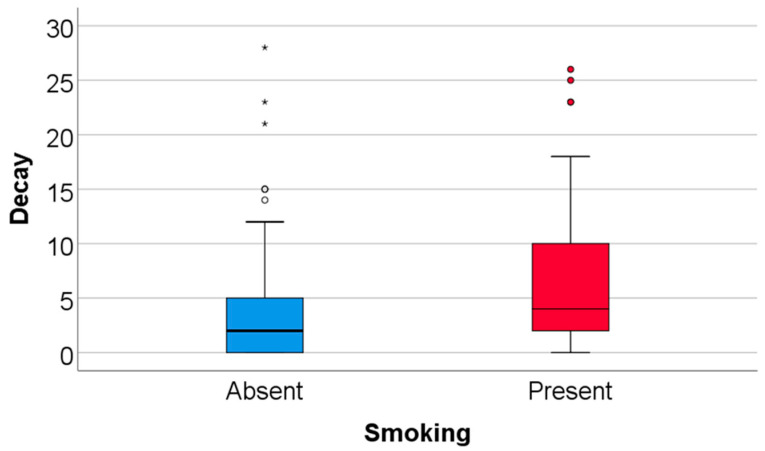
The correlation between caries index and smoking. * indicates extreme outliers (values located more than 3 interquartile ranges from the box), while open circles represent mild outliers (values between 1.5 and 3 interquartile ranges from the quartiles).

**Figure 4:**
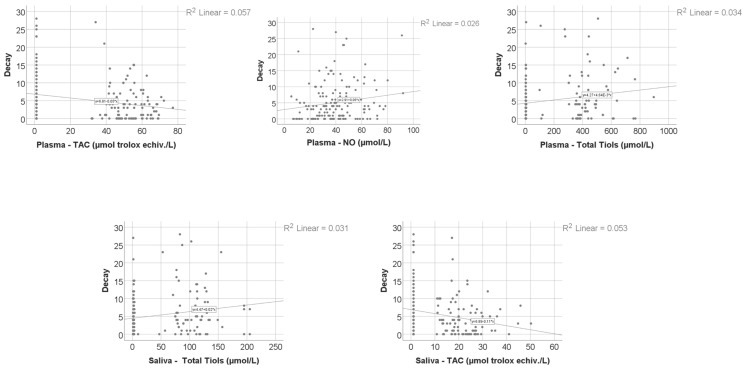
The correlation between caries index and oxidative stress markers.

Data from Table 8 and Figure 1, Figure 2, Figure 3 and Figure 4 show the distribution and correlations of decay index according to other investigated parameters. The results show the following significant differences: (i) Smokers had a significantly higher decay index (median = 4, IQR = 2–10) in comparison to non-smokers (median = 2, IQR = 0–5) (*p* < 0.001); (ii) plasmatic NO (*p* = 0.012, R = 0.192), plasmatic total thiols (*p* = 0.025, R = 0.173), salivary total thiols (*p* = 0.022, R = 0.184) and consumption frequencies of carbonated drinks with sweeteners (*p* < 0.001, R = 0.277), carbonated drinks with sugar (*p* < 0.001, R = 0.372), non-carbonated drinks with sugar (*p* < 0.001, R = 0.303), white bread/white flour products (*p* = 0.010, R = 0.187), croissants/muffins/biscuits (*p* = 0.006, R = 0.199), donuts (*p* = 0.013, R = 0.180) and pies or puddings (*p* = 0.040, R = 0.149) all had significant positive weak-to-moderate correlations with decay index; (iii) plasmatic CEL (*p* = 0.010, R = −0.196), salivary FruLys (*p* = 0.047, R = −0.155), salivary MG-H1 (*p* = 0.012, R = −0.194), salivary CML (*p* = 0.029, R = −0.170), salivary Arg (*p* = 0.001, R = −0.251), salivary Lys (*p* = 0.045, R = −0.156), plasma TAC (*p* = 0.018, R = −0.183), salivary TAC (*p* = 0.001, R = −0.260), salivary MDA (*p* = 0.002, R = −0.250) and consumption frequency of wholemeal bread (*p* = 0.012, R = −0.182) all had significant negative weak-to-moderate correlations with decay index. The decay index did not differ significantly according to snack frequency (Kruskal–Wallis test, *p* = 0.051), indicating a borderline, non-significant trend. Post hoc pairwise comparisons were conducted using the Mann–Whitney U test with Bonferroni correction. No significant differences were observed between the Daily and Many times/day groups (*p* = 1.000) or between the Daily and Rarely groups (*p* = 0.327). The comparison between Many times/day and Rarely yielded a nominally significant result after correction (*p* = 0.048), with participants reporting rare snack consumption showing a higher decay index (median = 4, IQR = 2–9) compared to those consuming snacks many times per day (median = 2.5, IQR = 0–5.25). Given that the overall test did not reach conventional statistical significance, these pairwise findings should be interpreted with caution.

Data from Table 9 show the univariable and multivariable linear regression models used to examine factors independently associated with decay index. The results according to the multivariable model show that the included variables were independently associated with decay index: (i) Higher salivary arginine was associated with a lower decay index (B = −2.091, 95% C.I. = −3.68–−0.49) (*p* = 0.011); (ii) consumption frequency of carbonated drinks with sugar was associated with a higher decay index (B = 0.61, 95% C.I. = 0.19–1.02) (*p* = 0.004); (iii) consumption frequency of non-carbonated drinks with sugar was associated with a higher decay index (B = 0.5, 95% C.I. = 0.034–0.982) (*p* = 0.036); (iv) consumption frequency of wholemeal bread was associated with a lower decay index (B = −0.442, 95% C.I. = −0.76–−0.11) (*p* = 0.008); (v) consumption frequency of croissants, muffins or biscuits was associated with a higher decay index (B = 0.54, 95% C.I. = 0.138–0.941) (*p* = 0.009); and (vi) consumption frequency of donuts was associated with a higher decay index (B = 0.784, 95% C.I. = 0.069–1.499) (*p* = 0.032).

Data from Table 10 show the multivariable step-wise forward linear regression model used to examine associations with decay index with age adjustment. In a univariable model, age was inversely associated with decay index (B = −0.189, 95% C.I. = −0.239–−0.139, *p* < 0.001). Using the same variables as the previous model in a step-wise forward multivariable linear regression model, consumption frequency of donuts (*p* = 0.015), non-carbonated drink with sugar (*p* = 0.007), wholemeal bread (*p* = 0.005) and croissants/muffins/biscuits (*p* = 0.034) remained independently associated with decay index while adjusting for age (*p* = 0.002), while salivary Arg level (*p* = 0.171) and consumption frequency of carbonated drinks with sugar (*p* = 0.152) were excluded in the final model due to lack of significance.

**Figure 5:**
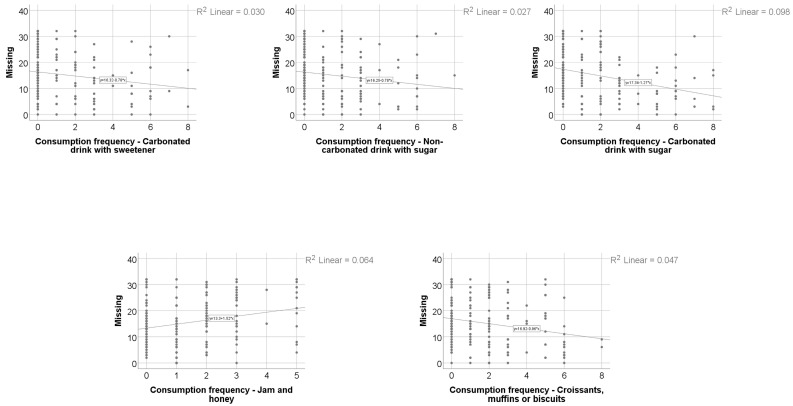
Correlation between missing index and diet.

Data from Table 11 and Figure 5 show the correlations and distribution of the missing index according to other investigated parameters. The results show the following significant differences: (i) Consumption frequency of jam/honey (*p* = 0.005, R = 0.204) had a significant weak positive correlation with the missing index, showing that patients that had higher values of the jam/honey consumption were significantly more associated with higher values of the missing index and vice versa; (ii) consumption frequencies of carbonated drink with sweetener (*p* = 0.026, R = −0.162), carbonated drinks with sugar (*p* < 0.001, R = −0.330), non-carbonated drinks with sugar (*p* = 0.002, R = −0.228) and croissants/muffins/biscuits (*p* = 0.004, R = −0.208) all had significant weak-to-moderate inverse correlations with the missing index, showing that patients that had lower values of the aforementioned parameters were significantly more associated with higher values of the missing index and vice versa.

Data from Table 12 shows the univariable and multivariable linear regression models used to examine factors independently associated with the missing index. The results according to the multivariable model show the following included variables were independently associated with the missing index: (i) Consumption frequency of carbonated drinks with sugar was associated with a lower missing index (B = −0.92, 95% C.I. = −1.65–−0.19) (*p* = 0.013); (ii) consumption frequency of croissants, muffins or biscuits was associated with a lower missing index (B = −0.79, 95% C.I. = 0.18–1.4) (*p* = 0.011); (iii) consumption frequency of jam or honey was associated with a higher missing index (B = 1.61, 95% C.I. = 0.81–2.42) (*p* < 0.001).

Correlation analysis revealed weak but statistically significant associations between the filled teeth index and salivary TOS (r = 0.194, *p* = 0.015), salivary NO (r = 0.173, *p* = 0.037), and frequency of jam/honey consumption (r = −0.150, *p* = 0.039).

However, when these variables were entered separately into univariable linear regression models, none demonstrated a statistically significant predictive effect on the filled teeth index. Specifically, salivary TOS showed β = 0.053 (95% C.I.: −0.069 to 0.174, *p* = 0.394), salivary NO showed β = −0.013 (95% C.I.: −0.079 to 0.053, *p* = 0.693), and jam/honey consumption showed β = −0.204 (95% C.I.: −0.478 to 0.070, *p* = 0.144).

These findings suggest that, although weak correlations were observed, the effect sizes were small and not robust in regression analysis. Additional details are provided in the Supplementary Material.

Data from Table 13 show the multivariable step-wise forward linear regression model used to examine associations with the missing index with age adjustment. In a univariable model, age was positively associated with the missing index (B = 0.374, 95% C.I. = 0.311–0.437, *p* < 0.001). Using the same variables as the previous model in a step-wise forward multivariable linear regression model, only the consumption frequency of jam or honey (*p* = 0.004) remained independently associated with the missing index while adjusting for age (*p* < 0.001), while the consumption frequencies of carbonated drinks with sweetener (*p* = 0.150), carbonated drinks with sugar (*p* = 0.345), non-carbonated drinks with sugar (*p* = 0.420) and croissants/muffins/biscuits (*p* = 0.519) were excluded from the model due to lack of significance. After adjustment for age, only consumption frequency of jam/honey remained independently associated with the missing index, while associations with sugar-sweetened beverages and refined carbohydrates lost statistical significance.

**Figure 6:**
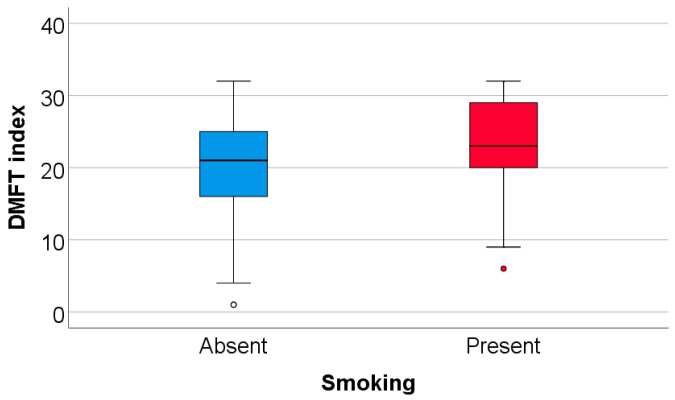
Comparison of DMFT index according to the existence of smoking.

**Figure 7:**
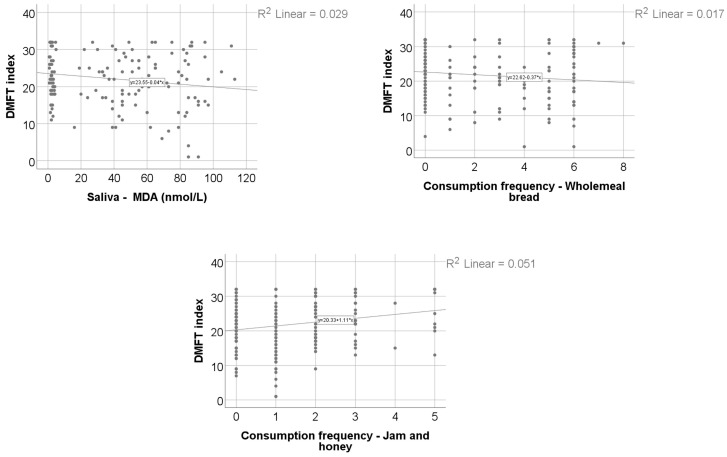
Comparison of DMFT index and diet.

Data from Table 14 and Figure 6 and Figure 7 show the correlations and distribution of the DMFT index according to other investigated parameters. The results show the following significant differences: (i) Smokers had a significantly higher value of DMFT index (median = 23, IQR = 19–29.5) in comparison to non-smokers (median = 21, IQR = 16–25.5) (*p* = 0.015); (ii) consumption frequency of jam/honey (*p* = 0.017, R = 0.174) had a significant weak positive correlation with the DMFT index; (iii) salivary MDA (*p* = 0.040, R = −0.165) and consumption frequency of wholemeal bread (*p* = 0.033, R = −0.156) both had significant weak inverse correlations with the DMFT index.

Data from Table 15 show the univariable and multivariable linear regression models used to examine factors independently associated with the DMFT index. The results according to the multivariable model show the following included variables were independently associated with the DMFT index: (i) Smoking status was associated with a higher DMFT index (B = 3.01, 95% C.I. = 0.81–5.2) (*p* = 0.007); (ii) salivary MDA was inversely associated with the DMFT index (B = −0.04, 95% C.I. = −0.07- −0.007) (*p* = 0.018); (iii) consumption frequency of jam or honey was associated with a higher DMFT index (B = 1.39, 95% C.I. = 0.67–2.11) (*p* < 0.001).

Data from Table 16 show the multivariable step-wise forward linear regression model used to examine associations with the DMFT index with age adjustment. In a univariable model, age was positively associated with the DMFT index (B = 0.135, 95% C.I. = 0.070–0.200, *p* < 0.001). Using the same variables as the previous model in a step-wise forward multivariable linear regression model, smoking status (*p* = 0.001), salivary MDA (*p* = 0.028), and consumption frequency of jam/honey (*p* = 0.001) remained independently associated with the DMFT index while adjusting for age (*p* < 0.001).

## 4. Discussion

Dental caries is widely recognized as a multifactorial condition shaped by the complex interplay between dietary habits, lifestyle behaviors such as smoking, and biochemical mechanisms including oxidative stress and advanced glycation end product (AGE) formation (Figure 1).

In addition to these interrelationships, aging represents a fundamental determinant of cumulative oral disease experience. Because caries indices such as DMFT and the missing component reflect lifetime disease burden, failure to account for age may lead to misleading or biologically implausible associations. Therefore, all regression models in the present study were additionally reconstructed with age adjustment, allowing a more accurate interpretation of independent associations.

Within this framework, we observed that higher consumption of sugar-rich and refined carbohydrate foods, as well as smoking, were consistently associated with increased decay and DMFT values, whereas wholemeal bread showed inverse associations. Several oxidative stress markers and salivary AGE-related biomarkers displayed significant associations with caries indices, suggesting that redox imbalance and altered protein glycation may accompany caries experience. After age adjustment, current dietary patterns remained independently associated with active decay, while age emerged as the dominant factor associated with tooth loss.

Similarly, for the DMFT index, smoking status, salivary MDA levels, and jam/honey consumption remained independently associated after age adjustment, whereas other dietary variables that showed weak associations in unadjusted analyses did not retain statistical significance. This comparison between unadjusted and age-adjusted models indicates that some observed associations were partially influenced by age-related variability, particularly for the missing component, while others remained robust independent associations across models.

The findings of the present study highlight the increasingly recognized biochemical interplay between dental caries and systemic pathways involving oxidative stress and advanced glycation end products (AGEs). Traditionally regarded as a localized infectious process driven by microbial dysbiosis and carbohydrate metabolism, dental caries is now understood to encompass broader metabolic alterations that reflect the host’s redox balance and glycation burden. The observed associations between caries severity and elevated oxidative stress markers, together with changes in the general pool of AGEs, suggest that carious lesions may not only be associated with microbial activity but also reflect underlying oxidative and glycation-mediated disturbances. These results align with emerging evidence that redox imbalance and non-enzymatic protein glycation have been proposed to be associated with inflammatory responses, tissue degradation, and modulation of the oral microenvironment, and may be involved in both the onset and progression of carious disease.

In the present study, the mean DMFT index was 21.89 ± 7.13 (median = 22), with the “Missing” component (M = 15.47 ± 8.72) predominating over the “Decayed” component (D = 5.20 ± 5.95). Compared with European data, our estimate exceeds the typical values reported for adults aged 35–44 years (mean 6.6–17.6; median ≈ 12.1) and approaches those observed in older adults (65–74 years), where DMFT values generally range between 14.7 and 25.5, with a median of ≈22. The high proportion of missing teeth reflects patterns observed in populations where tooth extraction remains a common treatment outcome. Considering that adult DMFT values range between 0 and 28 teeth, our mean of ≈22 indicates a substantial lifetime burden of caries and tooth loss.

### 4.1. Dietary Patterns and Caries Susceptibility

Our cohort exhibited frequent exposure to fermentable carbohydrates: 40.5% reported daily snacking, and 33.2% consumed white bread or other white flour products two to three times per day. Current evidence shows that the frequency of free-sugar or fermentable carbohydrate intake is more critical in caries development than total quantity. WHO and ADA recommendations emphasize limiting free sugars to below 5–10% of total energy intake to minimize caries risk (WHO, 2015 [34]; Moynihan & Kelly, 2014 [35]). Even when the intake of “typical sweets” and sugar-sweetened beverages is low, commonly consumed refined starches such as white bread can lower plaque pH and promote enamel demineralization. Rapidly digestible starches, prevalent in white flour-based foods, are associated with higher caries risk, whereas wholegrain alternatives are less cariogenic [35].

Although our participants reported very low consumption of both sugar-sweetened and “diet” soft drinks, which are frequently implicated in caries development, this pattern likely shifts explanatory weight toward refined starches and snack frequency as major contributors (Moynihan & Petersen, 2004 [36]). Reduced fruit consumption (e.g., apples or pears 2–4 times/week in 22.3% of respondents) also suggests suboptimal diet quality. Collectively, these dietary behaviors are consistent with the elevated cumulative DMFT index observed and are consistent with studies showing that lower intake of free sugars and refined carbohydrates is associated with reduced caries incidence [37,38,39,40,41,42,43,44,45,46].

### 4.2. Biochemical Markers: Oxidative Stress and Protein Glycation

The decay index showed significant correlations with several oxidative stress biomarkers and glycation-related parameters. Positive associations were noted with plasmatic nitric oxide and total thiols, while total antioxidant capacity (TAC) in both plasma and saliva correlated negatively with caries indices. These patterns suggest that individuals with higher caries scores tend to exhibit signs of oxidative imbalance, although the directionality or causal nature of this relationship cannot be inferred from the current data [47]. This observation aligns with previous reports describing elevated oxidative markers in the saliva and plasma of individuals with active lesions [48].

Interestingly, higher salivary malondialdehyde (MDA) levels were associated with slightly lower DMFT scores, which may reflect adaptive antioxidant mechanisms or improved oxidative regulation in individuals with better oral health [47].

Negative correlations between AGE-related biomarkers (CEL, CML, MG-H1, FruLys) and caries suggest that altered salivary protein glycation under oxidative conditions may modulate bacterial adhesion and biofilm formation [49,50]. Furthermore, higher salivary arginine levels were significantly associated with a reduction in the decay index, consistent with a potential protective role of arginine metabolism in maintaining plaque pH via the arginine deiminase system [51]. Together, these biochemical findings underscore the importance of redox and metabolic homeostasis in caries resistance. Although AGEs are often described as pro-inflammatory mediators in systemic disease, the interpretation of salivary AGE-related markers in the context of active caries is not straightforward. First, the salivary compartment reflects a dynamic balance between local production, protein turnover, and clearance, and may be strongly influenced by salivary flow rate and composition, which can vary with oral status and behaviors. Second, the measured AGE-related analytes likely represent only a subset of the broader glycation milieu and may not directly mirror tissue-level AGE accumulation or inflammatory signaling. Third, active caries and dietary adaptations may alter the availability and partitioning of glycated products between saliva, dental biofilm, and oral tissues. Therefore, the observed inverse correlations should be interpreted cautiously as associations rather than evidence of a protective effect; mechanistic conclusions require longitudinal and experimental studies that integrate microbial profiling, salivary flow/composition, and tissue-level AGE assessment.

The multivariable regression analysis further demonstrated that both biochemical parameters (notably salivary arginine) and multiple dietary variables were independently associated with decay index. When age-adjusted models were constructed, the overall direction and significance of most dietary variables remained unchanged. Consumption frequencies of donuts, non-carbonated sugar-sweetened beverages, croissants/muffins/biscuits, and wholemeal bread retained their significant associations with the decay index, whereas salivary arginine and carbonated drinks with sugar lost statistical significance. These findings suggest that current dietary patterns are strongly associated with active caries indices, while some biochemical associations observed in unadjusted models may be partially explained by age-related variability.

### 4.3. Behavioral and Lifestyle Determinants

Smoking emerged as a strong behavioral correlate of oral health outcomes. Smokers exhibited significantly higher decay and DMFT indices than non-smokers, and regression analysis confirmed that smoking was associated with an approximately 3-unit higher DMFT score [52,53,54,55,56,57,58,59,60,61]. These findings align with previous reports that smoking has been associated with greater caries experience and tooth loss, potentially through mechanisms such as reduced salivary flow, impaired immune response, and enhanced colonization by cariogenic microorganisms [62,63].

Dietary habits were further associated with differences in DMFT index. Frequent consumption of jam or honey was associated with an approximately 1.4 unit higher DMFT index, consistent with the well-documented cariogenic role of free sugars. Conversely, higher intake of wholemeal bread was associated with lower caries experience, reinforcing the observed inverse association of complex, fiber-rich carbohydrates [35]. Regarding tooth loss, greater jam and honey consumption was positively associated with the missing index, which may reflect the cumulative impact of sugar exposure on caries progression and extractions [38,64,65,66,67,68,69,70,71,72,73]. Meanwhile, inverse correlations between missing index and consumption of carbonated drinks or refined snacks likely indicate reverse causality, where individuals with extensive tooth loss modify their diet due to chewing limitations [36,74,75].

In unadjusted analyses, inverse associations were observed between sugar-sweetened beverage consumption and the missing index, a finding that appears counterintuitive. However, after adjustment for age, these associations were no longer statistically significant, and age emerged as the primary and strongest correlate of the missing index. This indicates that the inverse relationships observed in unadjusted models were driven by age-related confounding: Younger individuals reported higher consumption of sugary beverages but had accumulated fewer missing teeth due to shorter lifetime exposure, whereas older individuals exhibited greater tooth loss despite lower reported consumption.

### 4.4. General Interpretation

Given the cross-sectional design, these associations should be interpreted as observational and not indicative of causal relationships. Overall, our findings illustrate that dental caries and tooth loss are shaped by a complex interplay of lifestyle, dietary, and biochemical factors. Tobacco use, high-frequency intake of free sugars and refined carbohydrates, and oxidative stress imbalances are all associated with increased caries burden. In contrast, protective influences include greater antioxidant capacity, higher salivary arginine concentrations, and diets emphasizing whole grains. Importantly, smoking, salivary MDA, and jam/honey consumption remained independently associated with DMFT even after adjustment for age, suggesting that these factors are independently associated with cumulative caries experience beyond chronological aging, rather than confirming causal effects. These results reinforce the need for integrated preventive strategies that combine smoking cessation, dietary modification, and the promotion of antioxidant and arginine-rich diets to reduce the cumulative oral disease burden.

### 4.5. Limitations and Future Research

This study has several limitations that should be considered when interpreting the findings. The cross-sectional design prevents the establishment of temporal or causal relationships between caries status, oxidative stress markers, and glycation-related parameters; therefore, observed associations should not be interpreted as directional or mechanistic effects. Although the overall sample size exceeded the a priori calculated requirement, some biochemical parameters were available only for subgroups of participants due to biological sample availability, resulting in variable-specific missingness. Nevertheless, multivariable regression models retained sample sizes above the minimum required threshold, supporting adequate statistical power.

An additional methodological consideration relates to the saliva collection protocol. Salivary samples were obtained using the citric acid-stimulated collection (Salivette^®^ system), which may influence salivary flow rate and biochemical composition. Acid stimulation can modify protein concentration and potentially affect oxidative stress-related biomarkers. Although all samples were collected using a standardized and identical protocol across participants, thereby preserving internal comparability, the use of stimulated saliva may limit direct comparison with studies employing unstimulated whole saliva. This potential influence on biomarker concentration should therefore be considered when interpreting absolute salivary values.

Biomarker levels were assessed at a single time point, which may not fully capture dynamic fluctuations in redox balance or glycation processes. In addition, although age-adjusted models were constructed, residual confounding by unmeasured behavioral, metabolic, or environmental factors cannot be completely excluded. While dental hygiene frequency was recorded, detailed information regarding fluoride toothpaste use and history of professional dental cleaning was not collected; these represent important determinants of caries risk and may have influenced the observed associations.

Multiple correlation analyses were conducted across numerous biochemical and dietary variables. Given the exploratory and hypothesis-generating nature of these analyses, formal correction for multiple testing (e.g., Bonferroni adjustment) was not applied, as such procedures may increase the likelihood of type II error when evaluating biologically interrelated variables. Interpretation was therefore guided by effect size, biological plausibility, and consistency across multivariable regression models. Nevertheless, the possibility of type I error cannot be entirely excluded, and correlation-based findings should be interpreted with caution.

Future research should incorporate longitudinal designs to clarify temporal trends and causal pathways, include larger and more diverse cohorts, and integrate additional biochemical, microbiological, and clinical variables. Experimental or interventional studies may further elucidate the mechanistic links between oxidative imbalance, AGE accumulation, and caries activity.

## 5. Conclusions

This study demonstrates that dental caries and tooth loss are associated with the combined influence of lifestyle, dietary, and biochemical factors. The high DMFT values observed reflect a substantial cumulative burden of oral disease, which in this cohort was associated with frequent intake of free sugars and refined carbohydrates, oxidative imbalance, and smoking. In contrast, higher salivary arginine concentrations, greater antioxidant capacity, and wholegrain-rich diets showed protective associations. Importantly, these associations remained consistent after adjustment for age, indicating that dietary habits, smoking, and selected biochemical parameters are independently associated with caries experience beyond chronological aging. While causality cannot be inferred from the present cross-sectional design, these findings highlight the potential value of integrated preventive approaches that address modifiable lifestyle and dietary factors in the context of oral health promotion.

## Figures and Tables

**Table 1:** Baseline characteristics of the study population.

Parameter	Value
Gender (Female) (Nr., %) (N = 195)	126 (64.6%)
Age (Mean ± SD) (Median (IQR)) (N = 194)	50.55 ± 15.31, 53 (39–62.25)
BMI (Mean ± SD) (Median (IQR)) (N = 178)	29.64 ± 6.94, 28.75 (24.97–33.47)
Periodontal staging (Nr., %) (N = 170)	
Stage I	26 (15.3%)
Stage II	29 (17.1%)
Stage III	50 (29.4%)
Stage IV	37 (21.8%)
Gingivitis	11 (6.5%)
Healthy status	17 (10%)
Dental hygiene frequency (Nr., %) (N = 194)	
Rarely	28 (14.4%)
After every meal	3 (1.5%)
Once/day	61 (31.4%)
Twice/day	99 (51%)
Thrice/day	3 (1.5%)
Medical history—Comorbidities (Nr., %)	
Cardiovascular (N = 191)	76 (39.8%)
Hepatic (N = 190)	28 (14.7%)
Endocrine (N = 190)	31 (16.3%)
Diabetes (N = 192)	25 (13%)
Hematologic (N = 188)	9 (4.8%)
Tuberculosis (N = 191)	6 (3.1%)
Spasmophilia (N = 190)	9 (4.7%)
Epilepsy (N = 191)	4 (2.1%)
Asthma (N = 193)	17 (8.8%)
Radiotherapy/Chemotherapy (N = 183)	7 (3.8%)
Other (N = 191)	28 (14.7%)

**Table 2:** Description of the dental parameters analyzed in the study.

Parameter	Mean ± SD	Median (IQR)	Min	Max
Decay (N = 194)	5.2 ± 5.95	4 (1–7)	0	28
Missing (N = 194)	15.47 ± 8.72	15 (8–22)	0	32
DMFT index (N = 194)	21.89 ± 7.13	22 (17–27.25)	1	32

**Table 3:** Description of the plasma and salivary oxidative stress parameters analyzed in the study.

Plasma Oxidative Stress				
Parameter	Mean ± SD	Median (IQR)	Min	Max
TAC (N = 177)	34.35 ± 26.51	45.77 (1.09–55.79)	1	77
TOS (N = 178)	43.33 ± 19.14	41.95 (29.63–54.79)	6	88
OSI (N = 178)	17.06 ± 23.1	1.1 (1.09–36.81)	1	76
NO (N = 178)	37.95 ± 16.76	37.01 (27.19–45.83)	5	92
MDA (N = 178)	285 ± 227.67	359 (5.3–467)	2	779
Total thiols (N = 178)	157 ± 218.4	4.05 (3.08–375)	2	765
Salivary oxidative stress				
TAC (N = 165)	14.71 ± 12.19	16.54 (1.1–23.81)	1	50
TOS (N = 166)	8.09 ± 3.44	7.48 (5.51–9.75)	3	20
OSI (N = 168)	3.51 ± 3.54	1.1 (1.09–5.97)	1	18
NO (N = 165)	10.64 ± 7.15	8.6 (5.04–13.04)	3	32
MDA (N = 164)	38.39 ± 32.9	39 (2.93–64)	1	113
Total thiols (N = 164)	39.05 ± 54.58	2.53 (1.84–81)	1	205

**Table 4:** Measurement of plasma and salivary AGE parameters analyzed in the study.

Plasma AGEs (N = 181)				
Parameter	Mean ± SD	Median (IQR)	Min	Max
FruLys	337.51 ± 254.12	257.3 (140.2–435.7)	75.3	1299.8
Pyr	26.7 ± 7.67	28.39 (24.92–29.16)	5.2	56.86
MG-H1	35.06 ± 10.48	33.3 (31.45–36.67)	9.2	117.65
CEL	12.41 ± 2.72	13.34 (12.91–13.65)	6.2	21.8
CML	48.96 ± 8.91	48.23 (45.34–51.57)	11	128.1
Arg	5.18 ± 4	3.91 (2.67–6.87)	0.46	27.84
Lys	6.67 ± 2.65	6.05 (5.13–7.25)	1.08	21.62
Salivary AGEs (N = 170)				
FruLys	25.03 ± 5.25	22.7 (21.07–28.98)	20.31	49.37
Pyr	1.9 ± 0.65	1.71 (1.69–1.78)	1.4	7.24
MG-H1	4.04 ± 2.22	3.56 (2.63–4.73)	2.21	21.9
CEL	1.36 ± 0.6	1.25 (1.05–1.48)	0.95	7.54
CML	4.51 ± 1.71	4.22 (3.27–5.12)	2.52	12.8
Arg	0.62 ± 0.5	0.51 (0.25–0.87)	0.033	2.39
Lys	0.87 ± 0.9	0.62 (0.27–1.09)	0.011	5.24

**Table 5:** Distribution of the patients according to the existence of smoking.

Smoking (N = 197)	Nr.	Percentage
Absent	114	57.9%
Present	83	42.1%

**Table 6:** Distribution of the patients according to eating habits.

Consumption Frequency (Nr., %)									
Snacks (N = 195)		Daily		Many times/day		Rarely			
		79 (40.5%)		50 (25.6%)		66 (33.8%)			
Consumption frequency (Nr., %)									
Eating habit	Never	1–3/month	1/week	2–4/week	5–6/week	1/day	2–3/day	4–5/day	≥6/day
Carbonated drink with sweetener (N = 196)	118 (60.2%)	21 (10.7%)	16 (8.2%)	18 (9.2%)	3 (1.5%)	8 (4.1%)	8 (4.1%)	2 (1%)	2 (1%)
Carbonated drink with sugar (N = 197)	97 (49.2%)	26 (13.2%)	22 (11.2%)	21 (10.7%)	4 (2%)	9 (4.6%)	10 (5.1%)	4 (2%)	4 (2%)
Non-carbonated drink with sugar (N = 196)	108 (55.1%)	27 (13.8%)	19 (9.7%)	20 (10.2%)	5 (2.6%)	6 (3.1%)	9 (4.6%)	1 (0.5%)	1 (0.5%)
White bread and other white flour products (N = 196)	41 (20.9%)	9 (4.6%)	9 (4.6%)	11 (5.6%)	11 (5.6%)	40 (20.4%)	65 (33.2%)	6 (3.1%)	4 (2%)
Wholemeal bread (N = 195)	93 (47.7%)	14 (7.2%)	11 (5.6%)	15 (7.7%)	9 (4.6%)	19 (9.7%)	31 (15.9%)	2 (1%)	1 (0.5%)
Croissants, muffins or biscuits (N = 197)	83 (42.1%)	32 (16.2%)	33 (16.8%)	18 (9.1%)	4 (2%)	14 (7.1%)	11 (5.6%)	0 (0%)	2 (1%)
Pancakes/waffles (N = 195)	88 (45.1%)	66 (33.8%)	25 (12.8%)	13 (6.7%)	1 (0.5%)	2 (1%)	0 (0%)	0 (0%)	0 (0%)
Cream (N = 197)	139 (70.6%)	32 (16.2%)	14 (7.1%)	9 (4.6%)	1 (0.5%)	1 (0.5%)	0 (0%)	0 (0%)	1 (0.5%)
Milk chocolate (N = 196)	88 (44.9%)	41 (20.9%)	25 (12.8%)	26 (13.3%)	5 (2.6%)	9 (4.6%)	1 (0.5%)	0 (0%)	1 (0.5%)
Dark chocolate (N = 196)	109 (55.6%)	34 (17.3%)	22 (11.2%)	24 (12.2%)	1 (0.5%)	5 (2.6%)	1 (0.5%)	0 (0%)	0 (0%)
Chocolate bars (N = 196)	117 (59.7%)	35 (17.9%)	16 (8.2%)	19 (9.7%)	1 (0.5%)	7 (3.6%)	1 (0.5%)	0 (0%)	0 (0%)
Donuts (N = 195)	99 (50.8%)	55 (28.2%)	25 (12.8%)	10 (5.1%)	2 (1%)	4 (2.1%)	0 (0%)	0 (0%)	0 (0%)
Cakes (N = 197)	63 (32%)	64 (32.5%)	39 (19.8%)	22 (11.2%)	2 (1%)	6 (3%)	0 (0%)	1 (0.5%)	0 (0%)
Pies, puddings (N = 197)	91 (46.2%)	40 (20.3%)	39 (19.8%)	22 (11.2%)	0 (0%)	5 (2.5%)	0 (0%)	0 (0%)	0 (0%)
Jam and honey (N = 197)	75 (38.1%)	47 (23.9%)	35 (17.8%)	24 (12.2%)	2 (1%)	14 (7.1%)	0 (0%)	0 (0%)	0 (0%)
Ice cream (N = 197)	68 (34.5%)	44 (22.3%)	34 (17.3%)	30 (15.2%)	8 (4.1%)	13 (6.6%)	0 (0%)	0 (0%)	0 (0%)
Apples or pears (N = 197)	37 (18.8%)	25 (12.7%)	33 (16.8%)	44 (22.3%)	19 (9.6%)	34 (17.3%)	5 (2.5%)	0 (0%)	0 (0%)
Orange juice (N = 194)	90 (46.4%)	39 (20.1%)	23 (11.9%)	29 (14.9%)	4 (2.1%)	8 (4.1%)	1 (0.5%)	0 (0%)	0 (0%)

**Table 7:** Analyzed correlations of the DMFT index, decay index and missing index with age.

Correlation	*p* *
DMFT index × Age	<0.001, R = 0.284
Decay × Age	<0.001, R = −0.487
Missing × Age	<0.001, R = 0.649

* *p*-values correspond to Spearman’s rho correlation coefficients.

**Table 8:** Distribution and correlation of decay index according to other investigated parameters.

Smoking	Mean ± SD	Median (IQR)	*p* *
Absent (N = 109)	3.84 ± 4.96	2 (0–5)	<0.001
Present (N = 81)	6.74 ± 6.26	4 (2–10)	
Snack frequency	Mean ± SD	Median (IQR)	*p* **
Daily (N = 77)	4.7 ± 5.65	4 (0.5–7)	0.051
Many times/day (N = 46)	4.07 ± 5.23	2.5 (0–5.25)	
Rarely (N = 65)	6.22 ± 6.07	4 (2–9)	
Correlation		*p* ***	
Decay × Plasma—FruLys (N = 173)		0.538, R = −0.047	
Decay × Plasma—Pyr (N = 173)		0.965, R = 0.003	
Decay × Plasma—MG-H1 (N = 173)		0.500, R = −0.052	
Decay × Plasma—CEL (N = 173)		0.010, R = −0.196	
Decay × Plasma—CML (N = 173)		0.946, R = 0.005	
Decay × Plasma—Arg (N = 173)		0.624, R = −0.037	
Decay × Plasma—Lys (N = 173)		0.107, R = −0.123	
Decay × Saliva—FruLys (N = 165)		0.047, R = −0.155	
Decay × Saliva—Pyr (N = 165)		0.104, R = −0.127	
Decay × Saliva—MG-H1 (N = 165)		0.012, R = −0.194	
Decay × Saliva—CEL (N = 165)		0.950, R = −0.005	
Decay × Saliva—CML (N = 165)		0.029, R = −0.170	
Decay × Saliva—Arg (N = 165)		0.001, R = −0.251	
Decay × Saliva—Lys (N = 165)		0.045, R = −0.156	
Decay × Plasma—TAC (N = 167)		0.018, R = −0.183	
Decay × Plasma—TOS (N = 168)		0.087, R = 0.132	
Decay × Plasma—OSI (N = 168)		0.391, R = 0.067	
Decay × Plasma—NO (N = 168)		0.012, R = 0.192	
Decay × Plasma—MDA (N = 168)		0.126, R = −0.119	
Decay × Plasma—Total thiols (N = 168)		0.025, R = 0.173	
Decay × Saliva—TAC (N = 157)		0.001, R = −0.260	
Decay × Saliva—TOS (N = 157)		0.246, R = 0.093	
Decay × Saliva—OSI (N = 159)		0.054, R = 0.153	
Decay × Saliva—NO (N = 146)		0.324, R = 0.082	
Decay × Saliva—MDA (N = 156)		0.002, R = −0.250	
Decay × Saliva—Total thiols (N = 155)		0.022, R = 0.184	
Decay × Consumption—Carbonated drink with sweetener (N = 189)		<0.001, R = 0.277	
Decay × Consumption—Carbonated drink with sugar (N = 190)		<0.001, R = 0.372	
Decay × Consumption—Non-carbonated drink with sugar (N = 189)		<0.001, R = 0.303	
Decay × Consumption—White bread/white flour products (N = 189)		0.010, R = 0.187	
Decay × Consumption—Wholemeal bread (N = 188)		0.012, R = −0.182	
Decay × Consumption—Croissants/muffins/biscuits (N = 190)		0.006, R = 0.199	
Decay × Consumption—Pancakes/waffles (N = 188)		0.116, R = 0.115	
Decay × Consumption—Cream (N = 190)		0.980, R = −0.002	
Decay × Consumption—Milk chocolate (N = 189)		0.168, R = 0.101	
Decay × Consumption—Dark chocolate (N = 189)		0.740, R = −0.024	
Decay × Consumption—Chocolate bars (N = 189)		0.763, R = 0.022	
Decay × Consumption—Donuts (N = 188)		0.013, R = 0.180	
Decay × Consumption—Cakes (N = 190)		0.096, R = 0.121	
Decay × Consumption—Pies/puddings (N = 190)		0.040, R = 0.149	
Decay × Consumption—Jam/honey (N = 190)		0.126, R = −0.111	
Decay × Consumption—Ice cream (N = 190)		0.683, R = 0.030	
Decay × Consumption—Apples/pears (N = 190)		0.153, R = −0.104	
Decay × Consumption—Orange juice (N = 187)		0.888, R = 0.010	

* Mann–Whitney U test, ** Kruskal–Wallis H test, *** Spearman’s rho correlation coefficient.

**Table 9:** Univariable and multivariable linear regression models examining variables associated with the decay index.

Parameter	Univariable		Multivariable (N = 157) *		
	B (95% C.I.)	*p*	B (95% C.I.)	*p*	VIF
Smoking	2.89 (1.29–4.5)	<0.001	-	-	-
Plasma—CEL	−0.12 (−0.44–0.18)	0.426	-	-	-
Saliva—FruLys	−0.12 (−0.30–0.05)	0.161	-	-	-
Saliva—MG-H1	−0.41 (−0.80–−0.01)	0.041	-	-	-
Saliva—CML	−0.57 (−1.1–−0.05)	0.031	-	-	-
Saliva—Arg	−2.66 (−4.42–−0.89)	0.003	−2.09 (−3.68–−0.49)	0.011	1.052
Saliva—Lys	−1.25 (−2.29–−0.20)	0.019	-	-	-
Plasma—TAC	−0.05 (−0.08–−0.02)	0.002	-	-	-
Plasma—NO	0.05 (0.004–0.108)	0.035	-	-	-
Plasma—Total thiols	0.005 (0.001–0.008)	0.018	-	-	-
Saliva—TAC	−0.11 (−0.18–−0.03)	0.004	-	-	-
Saliva—MDA	−0.03(−0.05–−0.004)	0.025	-	-	-
Saliva—Total thiols	0.02 (0.002–0.035)	0.027	-	-	-
Consumption—Carbonated drink with sweetener	0.94 (0.53–1.36)	<0.001	-	-	-
Consumption—Carbonated drink with sugar	1.10 (0.74–1.47)	<0.001	0.61 (0.19–1.02)	0.004	1.399
Consumption—Non-carbonated drink with sugar	1.00 (0.55–1.44)	<0.001	0.50 (0.03–0.98)	0.036	1.281
Consumption—White bread/white flour products	0.34 (−0.002–0.684)	0.051	-	-	-
Consumption—Wholemeal bread	−0.52 (−0.86–−0.18)	0.003	−0.44 (−0.76–−0.11)	0.008	1.089
Consumption—Croissants/muffins/biscuits	0.84 (0.42–1.26)	<0.001	0.54 (0.13–0.94)	0.009	1.106
Consumption—Donuts	1.29 (0.53–2.05)	0.001	0.78 (0.07–1.5)	0.032	1.109
Consumption—Pies/puddings	0.71 (0.008–1.411)	0.047	-	-	-

* Multivariable step-wise forward approach linear regression model, F (6,150) = 12.834, *p* < 0.001, adjusted R^2^ = 0.313, Durbin Watson score = 1.882.

**Table 10:** Age-adjusted multivariable step-wise linear regression model of factors associated with the decay index.

Parameter	B (95% C.I.)	*p*	VIF
Age	−0.092 (−0.151–−0.033)	0.002	1.230
Consumption frequency—Donuts	0.897 (0.181–1.612)	0.015	1.092
Consumption frequency—Non-carbonated drink with sugar	0.668 (0.189–1.147)	0.007	1.133
Consumption frequency—Wholemeal bread	−0.506 (−0.856–−0.155)	0.005	1.036
Consumption frequency—Croissants, muffins or biscuits	0.444 (0.035–0.852)	0.034	1.105

F (5,111) = 12.364, *p* < 0.001, adjusted R^2^ = 0.329, Durbin Watson score = 1.770. Excluded variables: smoking, salivary—MG-H1/CML/Arg/Lys/TAC/MDA/total thiols, plasma—TAC/NO/total thiols, consumption frequency—carbonated drink with sweetener/carbonated drink with sugar/pies or puddings.

**Table 11:** Distribution and correlation of missing index according to other investigated parameters.

Smoking	Mean ± SD	Median (IQR)	*p* *
Absent (N = 109)	15.28 ± 8.44	15 (8–21)	0.880
Present (N = 81)	15.65 ± 9.05	15 (8–23)	
Snack frequency	Mean ± SD	Median (IQR)	*p* **
Daily (N = 77)	16.16 ± 9.18	17 (8.5–23.5)	0.531
Many times/day (N = 46)	15.54 ± 9.24	14 (8–22.75)	
Rarely (N = 65)	14.43 ± 7.78	15 (7.5–19)	
Correlation		*p* ***	
Missing × Plasma—FruLys (N = 173)		0.357, R = 0.070	
Missing × Plasma—Pyr (N = 173)		0.271, R = −0.084	
Missing × Plasma—MG-H1 (N = 173)		0.402, R = 0.064	
Missing × Plasma—CEL (N = 173)		0.164, R = 0.106	
Missing × Plasma—CML (N = 173)		0.117, R = 0.120	
Missing × Plasma—Arg (N = 173)		0.501, R = 0.051	
Missing × Plasma—Lys (N = 173)		0.116, R = 0.120	
Missing × Saliva—FruLys (N = 165)		0.821, R = 0.018	
Missing × Saliva—Pyr (N = 165)		0.231, R = 0.094	
Missing × Saliva—MG-H1 (N = 165)		0.077, R = 0.138	
Missing × Saliva—CEL (N = 165)		0.764, R = 0.024	
Missing × Saliva—CML (N = 165)		0.836, R = −0.016	
Missing × Saliva—Arg (N = 165)		0.158, R = 0.110	
Missing x Saliva—Lys (N = 165)		0.868, R = −0.013	
Missing × Plasma—TAC (N = 167)		0.317, R = 0.078	
Missing × Plasma—TOS (N = 168)		0.325, R = 0.076	
Missing × Plasma—OSI (N = 168)		0.654, R = 0.035	
Missing × Plasma—NO (N = 168)		0.852, R = −0.014	
Missing × Plasma—MDA (N = 168)		0.665, R = −0.034	
Missing × Plasma—Total thiols (N = 168)		0.692, R = 0.031	
Missing × Saliva—TAC (N = 157)		0.782, R = 0.022	
Missing × Saliva—TOS (N = 157)		0.275, R = −0.088	
Missing × Saliva—OSI (N = 159)		0.977, R = −0.002	
Missing × Saliva—NO (N = 146)		0.786, R = −0.023	
Missing × Saliva—MDA (N = 156)		0.470, R = −0.058	
Missing × Saliva—Total thiols (N = 155)		0.511, R = 0.053	
Missing × Consumption—Carbonated drink with sweetener (N = 189)		0.026, R = −0.162	
Missing × Consumption—Carbonated drink with sugar (N = 190)		<0.001, R = −0.330	
Missing × Consumption—Non-carbonated drink with sugar (N = 189)		0.002, R = −0.228	
Missing × Consumption—White bread/white flour products (N = 189)		0.738, R = 0.024	
Missing × Consumption—Wholemeal bread (N = 188)		0.602, R = −0.038	
Missing × Consumption—Croissants/muffins/biscuits (N = 190)		0.004, R = −0.208	
Missing × Consumption—Pancakes/waffles (N = 188)		0.360, R = −0.067	
Missing × Consumption—Cream (N = 190)		0.934, R = −0.006	
Missing × Consumption—Milk chocolate (N = 189)		0.122, R = −0.113	
Missing × Consumption—Dark chocolate (N = 189)		0.206, R = 0.092	
Missing × Consumption—Chocolate bars (N = 189)		0.677, R = −0.031	
Missing × Consumption—Donuts (N = 188)		0.528, R = −0.046	
Missing × Consumption—Cakes (N = 190)		0.363, R = −0.066	
Missing × Consumption—Pies/puddings (N = 190)		0.613, R = −0.037	
Missing × Consumption—Jam/honey (N = 190)		0.005, R = 0.204	
Missing × Consumption—Ice cream (N = 190)		0.625, R = −0.036	
Missing × Consumption—Apples/pears (N = 190)		0.610, R = 0.037	
Missing × Consumption—Orange juice (N = 187)		0.324, R = −0.073	

* Mann–Whitney U test, ** Kruskal–Wallis H test, *** Spearman’s rho correlation coefficient.

**Table 12:** Univariable and multivariable linear regression models examining variables independently associated with the missing index.

Parameter	Univariable		Multivariable (N = 188) *		
	B (95% C.I.)	*p*	B (95% C.I.)	*p*	VIF
Consumption—Carbonated drink with sweetener	−0.78 (−1.41–−0.14)	0.017	0.23 (−0.52–0.98)	0.541	1.645
Consumption—Carbonated drink with sugar	−1.26 (−1.82–−0.71)	<0.001	−0.92 (−1.65–−0.19)	0.013	1.897
Consumption—Non-carbonated drink with sugar	−0.77 (−1.44–−0.10)	0.024	−0.38 (−1.07–−0.3)	0.275	1.241
Consumption—Croissants/muffins/biscuits	−0.96 (−1.58–−0.33)	0.003	−0.79 (−1.4–−0.18)	0.011	1.099
Consumption—Jam/honey	1.52 (0.68–2.35)	<0.001	1.61 (0.81–2.42)	<0.001	1.070

* Multivariable linear regression model, F (5,182) = 8.432, *p* < 0.001, adjusted R^2^ = 0.166, Durbin Watson score = 2.228.

**Table 13:** Age-adjusted multivariable step-wise forward linear regression model examining factors independently associated with the missing index.

Parameter	B (95% C.I.)	*p*	VIF
Age	0.357 (0.295–0.419)	<0.001	1.030
Consumption frequency—Jam/honey	0.972 (0.312–1.632)	0.004	1.030

F (2,177) = 76.577, *p* < 0.001, adjusted R^2^ = 0.458, Durbin Watson score = 2.041. Excluded variables: consumption frequency—carbonated drink with sweetener/carbonated drink with sugar/non-carbonated drink with sugar/croissants, muffins or biscuits.

**Table 14:** Correlations and distribution of the DMFT index according to other investigated parameters.

Smoking	Mean ± SD	Median (IQR)	*p* *
Absent (N = 109)	20.63 ± 7.25	21 (16–25.5)	0.015
			U = 3507
Present (N = 81)	23.3 ± 6.6	23 (19–29.5)	Z = 2.425
			r = 0.176
Snack frequency	Mean ± SD	Median (IQR)	*p* **
Daily (N = 77)	22.18 ± 7.2	23 (17.5–27)	0.555
Many times/day (N = 46)	20.96 ± 7.7	20 (15.75–28)	
Rarely (N = 65)	21.88 ± 6.6	22 (16.5–27)	
Correlation		*p* ***	
DMFT × Plasma—FruLys (N = 173)		0.402, R = 0.064	
DMFT × Plasma—Pyr (N = 173)		0.247, R = −0.089	
DMFT × Plasma—MG-H1 (N = 173)		0.385, R = 0.066	
DMFT × Plasma—CEL (N = 173)		0.704, R = 0.029	
DMFT × Plasma—CML (N = 173)		0.159, R = 0.107	
DMFT × Plasma—Arg (N = 173)		0.225, R = 0.093	
DMFT × Plasma—Lys (N = 173)		0.121, R = 0.118	
DMFT × Saliva—FruLys (N = 165)		0.234, R = −0.093	
DMFT × Saliva—Pyr (N = 165)		0.680, R = 0.032	
DMFT × Saliva—MG-H1 (N = 165)		0.765, R = 0.023	
DMFT × Saliva—CEL (N = 165)		0.986, R = −0.001	
DMFT × Saliva—CML (N = 165)		0.149, R = −0.113	
DMFT × Saliva—Arg (N = 165)		0.383, R = −0.068	
DMFT × Saliva—Lys (N = 165)		0.214, R = −0.097	
DMFT × Plasma—TAC (N = 167)		0.281, R = −0.084	
DMFT × Plasma—TOS (N = 168)		0.166, R = 0.107	
DMFT × Plasma—OSI (N = 168)		0.133, R = 0.116	
DMFT × Plasma—NO (N = 168)		0.555, R = 0.046	
DMFT × Plasma—MDA (N = 168)		0.066, R = −0.142	
DMFT × Plasma—Total thiols (N = 168)		0.206, R = 0.098	
DMFT × Saliva—TAC (N = 157)		0.057, R = −0.152	
DMFT × Saliva—TOS (N = 157)		0.501, R = −0.054	
DMFT × Saliva—OSI (N = 159)		0.151, R = 0.114	
DMFT × Saliva—NO (N = 146)		0.722, R = −0.030	
DMFT × Saliva—MDA (N = 156)		0.040, R = −0.165	
DMFT × Saliva—Total thiols (N = 155)		0.159, R = 0.114	
DMFT × Consumption—Carbonated drink with sweetener (N = 189)		0.744, R = 0.024	
DMFT × Consumption—Carbonated drink with sugar (N = 190)		0.303, R = −0.075	
DMFT × Consumption—Non-carbonated drink with sugar (N = 189)		0.718, R = 0.026	
DMFT × Consumption—White bread/white flour products (N = 189)		0.343, R = 0.069	
DMFT × Consumption—Wholemeal bread (N = 188)		0.033, R = −0.156	
DMFT × Consumption—Croissants/muffins/biscuits (N = 190)		0.672, R = −0.031	
DMFT × Consumption—Pancakes/waffles (N = 188)		0.537, R = 0.045	
DMFT × Consumption—Cream (N = 190)		0.923, R = −0.007	
DMFT × Consumption—Milk chocolate (N = 189)		0.822, R = −0.017	
DMFT × Consumption—Dark chocolate (N = 189)		0.780, R = 0.020	
DMFT × Consumption—Chocolate bars (N = 189)		0.442, R = −0.056	
DMFT × Consumption—Donuts (N = 188)		0.381, R = 0.064	
DMFT × Consumption—Cakes (N = 190)		0.236, R = 0.086	
DMFT × Consumption—Pies/puddings (N = 190)		0.365, R = 0.066	
DMFT × Consumption—Jam/honey (N = 190)		0.017, R = 0.174	
DMFT × Consumption—Ice cream (N = 190)		0.546, R = −0.044	
DMFT × Consumption—Apples/pears (N = 190)		0.241, R = −0.085	
DMFT × Consumption—Orange juice (N = 187)		0.329, R = −0.072	

* Mann–Whitney U test, ** Kruskal–Wallis H test, *** Spearman’s rho correlation coefficient.

**Table 15:** Univariable and multivariable linear regression models examining factors independently associated with the DMFT index.

Parameter	Univariable		Multivariable (N = 152) *		
	B (95% C.I.)	*p*	B (95% C.I.)	*p*	VIF
Smoking	2.66 (0.64–4.68)	0.010	3.01 (0.81–5.2)	0.007	1.033
Saliva—MDA	−0.03(−0.07–−0.003)	0.033	−0.04(−0.07–−0.007)	0.018	1.018
Consumption—Wholemeal bread	−0.37 (−0.79–0.04)	0.076	-	-	-
Consumption—Jam/honey	1.11 (0.42–1.79)	0.002	1.39 (0.67–2.11)	<0.001	1.016

* Multivariable linear regression model, F (3,148) = 8.932, *p* < 0.001, adjusted R^2^ = 0.136, Durbin Watson score = 2.232.

**Table 16:** Age-adjusted multivariable step-wise forward linear regression model examining factors independently associated with the DMFT index.

Parameter	B (95% C.I.)	*p*	VIF
Age	0.139 (0.069–0.209)	<0.001	1.051
Smoking	3.692 (1.518–5.867)	0.001	1.052
Saliva—MDA	−0.036 (−0.068–−0.004)	0.028	1.039
Consumption frequency—Jam/honey	1.230 (0.517–1.943)	0.001	1.017

F (4,141) = 11.004, *p* < 0.001, adjusted R^2^ = 0.216, Durbin Watson score = 2.054.

## Data Availability

The original contributions presented in this study are included in the article/Supplementary Material. Further inquiries can be directed to the corresponding author.

## Supplementary Materials

The following supporting information can be downloaded at: https://www.mdpi.com/article/10.3390/nu18060923/s1, Supplementary information regarding filled teeth.

## Abbreviations

The following abbreviations are used in this manuscript:

AGEs	Advanced Glycation End Products
Arg	Arginine
BMI	Body Mass Index
CEL	*N*-ε-carboxyethyllysine
CI	Confidence Interval
CML	*N*-ε-carboxymethyllysine
DMFT	Decayed, Missing, and Filled Teeth
EDTA	Ethylenediaminetetraacetic Acid
FruLys	Fructosyl-Lysine
IQR	Interquartile Range
LC–MS/MS	Liquid Chromatography–Tandem Mass Spectrometry
Lys	Lysine
MDA	Malondialdehyde
MG-H1	Methylglyoxal-Derived Hydroimidazolone-1
NO	Nitric Oxide
NOx	Nitric Oxide Metabolites (nitrite/nitrate)
OSI	Oxidative Stress Index
ROS	Reactive Oxygen Species
SD	Standard Deviation
TAC	Total Antioxidant Capacity
TAR	Total Antioxidant Response
TBARSs	Thiobarbituric Acid Reactive Substances
TOS	Total Oxidative Status
