# Supplementary material for: Exploring the Triangle Between Oxidative Stress, Advanced Glycation End Products and Dental Caries in the Context of Diet and Lifestyle
Source: Nutrients. 2026 Mar 14;18(6):923. doi: 10.3390/nu18060923 (PMC13029232; doi:10.3390/nu18060923)
Supplement: Supplementary file 1 [file nutrients-18-00923-s001.zip › nutrients-4172743-supplementary.pdf]

Supplementary information regarding filled teeth

Median filled teeth was 0 teeth (IQR = 0-1), mean = 1.273 ± 2.77. Correlation analyses between filled teeth and the other parameters show that almost all variables were not significantly correlated with the number of filled teeth, as shown below, except for salivary-TOS (p=0.015, R=0.194), salivary-NO (p=0.037,R=0.173) and consumption frequency of jam and honey (p=0.039, R= -0.150).

Spearman's rho Correlation Coefficient

Filled teeth

	R	Sig. (2-tailed)
Filled teeth		
Plasma - FruLys [µg/ml]	-0.078	0.307
Plasma -Pyr [ng/ml]	0.033	0.662
Plasma -MG-H1 [ng/ml]	0.048	0.533
Plasma -CEL [ng/ml]	-0.041	0.596
Plasma -CML [ng/ml]	-0.007	0.928
Plasma -Arg [µg/ml]	-0.013	0.869
Plasma -Lys [µg/ml]	-0.032	0.678
Saliva - FruLys [ng/ml]	-0.053	0.499
Saliva - Pyr [ng/ml]	0.031	0.688
Saliva - MG-H1 [ng/ml]	0.019	0.807
Saliva - CEL [ng/ml]	-0.059	0.453
Saliva - CML [ng/ml]	0.036	0.65
Saliva - Arg [µg/ml]	-0.013	0.867
Saliva - Lys [µg/ml]	-0.001	0.988
Plasma - TAC (µmol trolox echiv./L)	-0.071	0.364
Plasma - TOS (µmol H2O2 echiv./L)	0.014	0.86
Plasma - OSI	0.032	0.685
Plasma - NO (µmol/L)	0.061	0.434

Plasma - MDA (nmol/L)	-0.03	0.704
Plasma - Total Tiols (µmol/L)	0.06	0.44
Saliva - TAC (µmol trolox echiv./L)	0.077	0.336
**Saliva - TOS (µmol H2O2 echiv./L)**	**0.194**	**0.015**
Saliva - OSI	-0.04	0.619
**Saliva - NO (µmol/L)**	**0.173**	**0.037**
Saliva - MDA (nmol/L)	-0.008	0.921
Saliva - Total Tiols (µmol/L)	-0.012	0.885
Consumption frequency - Carbonated drink with sweetener	0.014	0.847
Consumption frequency - Carbonated drink with sugar	-0.045	0.539
Consumption frequency - Non-carbonated drink with sugar	-0.042	0.57
Consumption frequency - White bread and other white flour products	-0.119	0.104
Consumption frequency - Wholemeal bread	0.038	0.603
Consumption frequency - Croissants, muffins or biscuits	-0.07	0.34
Consumption frequency - Pancakes or waffles	-0.093	0.206
Consumption frequency - Cream	-0.02	0.784
Consumption frequency - Milk chocolate	0.091	0.214
Consumption frequency - Dark chocolate	-0.039	0.59
Consumption frequency - Chocolate bars	0.055	0.454
Consumption frequency - Donuts	-0.125	0.087
Consumption frequency - Cakes	0.084	0.252
Consumption frequency - Pies, puddings	-0.056	0.443
**Consumption frequency - Jam and honey**	**-0.15**	**0.039**
Consumption frequency - Ice cream	0.057	0.438
Consumption frequency - Apples or pears	0.01	0.892
Consumption frequency - Orange juice	-0.004	0.962

However, due to the small significantly influence of each of the three mentioned variables, when introduced in a univariable linear regression model, all of the three variables showed no significant prediction:

- Saliva – TOS – Beta – 0.053 (95% C.I. = -0.069 – 0.174), p=0.394
- Saliva – NO – Beta - -0.013 (95% C.I. = -0.079 – 0.053), p=0.693
- Consumption – Jam&Honey – Beta - -0.204 (95% C.I. = -0.478-0.070), p=0.144.

Forced-entry multivariable linear regression model using smoking as confounding variable for the decay index (using significant predictors in final step-wise model)

Model Summary[b]

					Change Statistics					
Model	R	R Square	Adjusted R Square	Std. Error of the Estimate	R Square Change	F Change	df1	df2	Sig. F Change	Durbin-Watson
1	0.585[a]	0.342	0.311	4.623	0.342	10.988	7	148	0.000	0.041

a. Predictors: (Constant), Consumption frequency - Donuts, Saliva - Arg [μg/ml], Consumption frequency - Non-carbonated drink with sugar, Consumption frequency - Wholemeal bread, Smoking, Consumption frequency - Croissants, muffins or biscuits, Consumption frequency - Carbonated drink with sugar

b. Dependent Variable: Decay

ANOVA[a]

Model		Sum of Squares	df	Mean Square	F	Sig.
1	Regression	1644.197	7	234.885	10.988	0.000[b]
	Residual	3163.720	148	21.376		
	Total	4807.917	155			

a. Dependent Variable: Decay

b. Predictors: (Constant), Consumption frequency - Donuts, Saliva - Arg [μg/ml], Consumption frequency - Non-carbonated drink with sugar, Consumption frequency - Wholemeal bread, Smoking, Consumption frequency - Croissants, muffins or biscuits, Consumption frequency - Carbonated drink with sugar

Model		Unstandardized Coefficients			95.0% Confidence Interval for B		Collinearity Statistics	
		B	Std. Error	Sig.	Lower Bound	Upper Bound	Tolerance	VIF
1	(Constant)	3.701	0.918	0.000	1.887	5.516		
	Smoking	0.731	0.811	0.369	-0.871	2.333	0.862	1.160
	Saliva - Arg [μg/ml]	-1.848	0.798	0.022	-3.425	-0.270	0.894	1.119
	Consumption frequency - Carbonated drink with sugar	0.546	0.210	0.010	0.131	0.961	0.656	1.526
	Consumption frequency - Non-carbonated drink with sugar	0.522	0.231	0.026	0.064	0.979	0.770	1.298
	Consumption frequency - Wholemeal bread	-0.407	0.158	0.011	-0.720	-0.094	0.920	1.087
	Consumption frequency - Croissants, muffins or biscuits	0.522	0.195	0.008	0.137	0.907	0.904	1.106
	Consumption frequency - Donuts	0.715	0.348	0.041	0.028	1.402	0.902	1.108

Forced-entry multivariable linear regression model using smoking as a confounding variable for the decay index (using all significant predictors in univariable models)

Model Summary[b]

Model	R	R Square	Adjusted R Square	Std. Error of the Estimate	Change Statistics: R Square Change	F Change	df1	df2	Sig. F Change	Durbin-Watson
1	0.630[a]	0.396	0.284	4.514	0.396	3.524	19	102	0.000	0.083

a. Predictors: (Constant), Consumption frequency - Pies, puddings, Consumption frequency - Non-carbonated drink with sugar, Saliva - MG-H1 [ng/ml], Consumption frequency - Wholemeal bread, Consumption frequency - Croissants, muffins or biscuits, Smoking, Saliva - MDA (nmol/L), Saliva - Arg [µg/ml], Consumption frequency - Carbonated drink with sweetener, Consumption frequency - Donuts, Consumption frequency - White bread and other white flour products, Saliva - CML [ng/ml], Plasma - NO (µmol/L), Consumption frequency - Carbonated drink with sugar, Saliva - Lys [µg/ml], Plasma - Total Tiols (µmol/L), Saliva - TAC (µmol trolox echiv./L), Saliva - Total Tiols (µmol/L), Plasma - TAC (µmol trolox echiv./L)

b. Dependent Variable: Decay

ANOVA[a]

Model		Sum of Squares	df	Mean Square	F	Sig.
1	Regression	1364.443	19	71.813	3.524	0.000[b]
	Residual	2078.377	102	20.376		
	Total	3442.820	121			

a. Dependent Variable: Decay

b. Predictors: (Constant), Consumption frequency - Pies, puddings, Consumption frequency - Non-carbonated drink with sugar, Saliva - MG-H1 [ng/ml], Consumption frequency - Wholemeal bread, Consumption frequency - Croissants, muffins or biscuits, Smoking, Saliva - MDA (nmol/L), Saliva - Arg [µg/ml], Consumption frequency - Carbonated drink with sweetener, Consumption frequency - Donuts, Consumption frequency - White bread and other white flour products, Saliva - CML [ng/ml], Plasma - NO (µmol/L), Consumption frequency - Carbonated drink with sugar, Saliva - Lys [µg/ml], Plasma - Total Tiols (µmol/L), Saliva - TAC (µmol trolox echiv./L), Saliva - Total Tiols (µmol/L), Plasma - TAC (µmol trolox echiv./L)

Model		Unstandardized Coefficients			95.0% Confidence Interval for B		Collinearity Statistics	
		B	Std. Error	Sig.	Lower Bound	Upper Bound	Tolerance	VIF
1	(Constant)	5.380	3.510	0.128	-1.583	12.343		
	Smoking	0.373	0.998	0.709	-1.607	2.353	0.708	1.413
	Saliva - MG-H1 [ng/ml]	-0.284	0.224	0.208	-0.728	0.160	0.679	1.472
	Saliva - CML [ng/ml]	-0.072	0.376	0.849	-0.817	0.674	0.453	2.207
	Saliva - Arg [µg/ml]	-1.790	1.110	0.110	-3.991	0.412	0.530	1.885
	Saliva - Lys [µg/ml]	0.291	0.816	0.722	-1.327	1.909	0.390	2.561
	Plasma - TAC (µmol trolox echiv./L)	-0.016	0.046	0.729	-0.107	0.075	0.107	9.389
	Plasma - NO (µmol/L)	-0.005	0.032	0.888	-0.069	0.060	0.520	1.921
	Plasma - Total Tiols (µmol/L)	-0.002	0.004	0.653	-0.010	0.006	0.163	6.146
	Saliva - TAC (µmol trolox echiv./L)	0.041	0.072	0.572	-0.102	0.184	0.200	4.998
	Saliva - MDA (nmol/L)	-0.021	0.025	0.402	-0.071	0.029	0.242	4.127
	Saliva - Total Tiols (µmol/L)	-0.003	0.016	0.844	-0.036	0.029	0.180	5.549
	Consumption frequency - Carbonated drink with sweetener	0.066	0.295	0.822	-0.518	0.651	0.566	1.767
	Consumption frequency - Carbonated drink with sugar	0.528	0.277	0.059	-0.021	1.078	0.489	2.044
	Consumption frequency - Non-carbonated drink with sugar	0.570	0.275	0.041	0.025	1.115	0.710	1.408
	Consumption frequency - White bread and other white flour products	0.140	0.203	0.491	-0.262	0.543	0.670	1.493
	Consumption frequency - Wholemeal bread	-0.373	0.204	0.071	-0.778	0.032	0.707	1.415
	Consumption frequency - Croissants, muffins or biscuits	0.403	0.234	0.088	-0.061	0.867	0.729	1.372
	Consumption frequency - Donuts	0.877	0.430	0.044	0.025	1.729	0.668	1.498
	Consumption frequency - Pies, puddings	0.294	0.433	0.499	-0.566	1.153	0.656	1.524
